# The Medical and Surgical Reporter, Philadelphia, Penn.

**Published:** 1858-11

**Authors:** 


THE MEDICAL AND SURGICAL REPORTER, PHILADELPHIA, PA.

This valuable Journal will hereafter be published in the weekly form, for which we return our thanks to the editors, as we honestly believe that just such a journal as we think they will make it, was a desideratum, and this is saying a good deal for a Medical Journal.

The editors remark in their Salutatory, that "It is rather remarkable that, with our world-wide reputation as a reading people, there is but one weekly medical journal in this country, and that not at the seat of medical education and literature; while in Great Britain and on the continent they are common in all the large cities. In the city of London alone there are several weeklies, published, too, at a subscription-price of nearly eight dollars a year; while in Paris there are medical journals published *three times* a week, besides others published weekly.

"To sum up:—We propose to meet a want in medical periodical literature; to perpetuate much valuable medical instruction that is now in a measure lost to the profession; to give additional interest and importance to our medical society meetings; and finally, to encourage a home medical literature by paying liberally for material with which to enrich our pages."

This enterprise deserves, and will, we are sure, receive the support of the profession.

Address Editors of the Medical and Surgical Reporter, Philadelphia, P. O. Box 1422, with $3, *invariably* accompanying the order, which amount is really not over *half* what should be the subscription, to carry out fully the objects of the projectors.

